# Patents on Surgical Instruments

**Published:** 1876-08

**Authors:** 


*Patents on Surgical Instruments.*—In our opinion the time has come when the code of ethics should be revised so as to allow physicians and surgeons the right to take letters patent upon discoveries, etc., in surgical mechanics. There is no sense in suppressing the mechanical skill of the profession under the operations of a false estimation of the ethics. Open wide the door, and let surgery receive the aid of medical inventive genius, as have every other department of mechanics. G.

